# Fine mapping and candidate gene analysis of *qTAC8*, a major quantitative trait locus controlling tiller angle in rice (*Oryza sativa* L.)

**DOI:** 10.1371/journal.pone.0178177

**Published:** 2017-05-25

**Authors:** Jiwai He, Gaoneng Shao, Xiangjin Wei, Fenglin Huang, Zhonghua Sheng, Shaoqing Tang, Peisong Hu

**Affiliations:** 1 State Key Laboratory of Rice Biology, China National Rice Research Institute, Hangzhou, China; 2 Hunan Rice Research Institute, Hunan Academy of Agricultural Science, Changsha, China; Institute of Genetics and Developmental Biology Chinese Academy of Sciences, CHINA

## Abstract

Rice tiller angle is an important agronomic trait that contributes to crop production and plays a vital role in high yield breeding. In this study, a recombinant inbred line (RIL) population derived from the cross of a glabrous *tropical japonica* rice D50 and an *indica* rice HB277, was used to investigate quantitative trait loci (QTLs) controlling rice tiller angle. Two major QTLs, *qTAC8* and *qTAC9*, were detected. While *qTAC9* mapped with a previously identified gene (*TAC1*), using a BC_2_F_2_ population *qTAC8* was mapped to a 16.5 cM region between markers RM7049 and RM23175. Position of *qTAC8* was narrowed to a 92 kb DNA region by two genetic segregating populations. Finally, one opening reading frame (ORF) was regarded as a candidate gene according to genomic sequencing and qRT-PCR analysis. In addition, a set of four near isogenic lines (NILs) were created to investigate the genetic relationship between those two QTLs, and one line carrying *qTAC8* and *qTAC9* presented additive effect of tiller angle, suggesting that these QTLs are involved in different genetic pathways. Our results provide a foundation for the cloning of *qTAC8* and genetic improvement of the rice plant architecture.

## Introduction

Rice (*Oryza sativa* L.) is one of the most important food crops in China and the world. It is the most effective safeguard for food security and agricultural sustainable development through high-yielding rice breeding. Ideotype breeding strategy is an important approach to increase grain yield potential in rice breeding. Tiller angle, the angle between the main culm and its side tillers [1], is a decisive factor for building ideal plant architecture, whereby neither spread-out rice nor compact type rice is beneficial for grain production [2]. With a spread-out architecture, plants can decrease humidity and escape from some diseases, but they occupy too much space and increase shading and lodging, consequently decreasing photosynthetic efficiency and grain yield per unit area. On the other hand, compact plants prejudice in capturing light and prevention of plant diseases and insect pests, thus appropriate tiller angle is beneficial for improving rice production [3, 4]. Although rice tiller angle has long attracted attention of breeders due to the significant contribution to plant architecture and yield potential, the genetic mechanisms determining its characteristics are not fully understood.

Rice tiller angle has been recognized as a complex quantitative trait, which is not only controlled by genetic factors but also greatly influenced by environmental conditions, such as light intensity, climate, soil, planting density, watering and fertilizing [4]. In the past two decades, a number of QTLs for tiller angle have been identified on chromosomes 1, 2, 5, 7, 8, 9, 11, 12 in various mapping populations of rice. Using an F_2_ population helped to identify three major genes controlling tiller angle [5]. A major QTL (*ta9*) on chromosome 9 flanked by RZ228 to RG667 together with other four QTLs (*QTa1*, *QTa2*, *QTa3*, *QTa8*) were separated in a F_2:4_ genetic segregating population generated from a cross between Lemont and Teqing [6]. In addition, a doubled haploid population generated from a cross between Zhaiyeqing 8 (loose plant architecture) and Jingxi 7 (compact plant architecture) was used and a total of three controlling tiller angle QTLs named *qTA-9a*, *qTA-9b*, *qTA-12*, that account for 22.7%, 11.9% and 20.9% of the variance, respectively, were detected [7]. Moreover, two major QTLs, *qTA8-2* and *qTA9-2* were determined in a recombinant inbred line population derived from Xieqingzao B/Miyang 46, and no G×E interaction effect was detected for the additive effect of these two QTLs [1]. Five major QTLs including *qTA-9*, *qTA-2*, *qTA-7a*, *qTA-7b* and *qTA-11* for tiller angle were present in a RIL population from a cross between Asominori and IR24. Then a major QTL, *qTA-9*, was singled out in a 15 cM region between RFLP markers C609 and C508 by using a CSSLs population [8]. To date, only two major QTLs for tiller angle have been cloned. One is *TAC1*, a major QTL for tiller angle was isolated by using a large F_2_ population that derives from the cross between *indica* rice IR24, and an introgression line IL55. *TAC1* harbors three introns in its coding region and a fourth 1.5 kb intron in the 3’ untranslated region, and encodes a 259-amino-acid unknown protein. A mutation in the 3’ splicing site of the fourth intron from ‘AGGA’ to ‘GGGA’ decreases its transcript levels, resulting in compact plant architecture. *tac1* has also been extensively utilized in densely planted rice [4]. The second cloned QTL for tiller angle corresponds to *PROG1* (*PROSTRATE GROWTH 1*), a semi-dominant gene encoding a newly identified Cys2-His2 zinc-finger transcription factor located on chromosome 7. *PROG1* is predominantly expressed in the axillary meristems and the site of tiller bud formation, and disrupting the *prog1* function and inactivate *prog1* expression can lead to erect growth, increasing grain number and higher grain yield in cultivated rice [9, 10]. Dong identified three major QTLs (*TAC3*, *DWARF2* (*D2*) and *TAC1*) controlling tiller angles by genome-wide association studies. *TAC3* encodes a conserved hypothetical protein of 152 amino acids that is preferentially expressed in the tiller base[11].

Previous reports indicate that the tiller angle is not only affected by QTLs but also could be controlled by single genes following the Mendel’s genetics. Thus far, several genes controlling tiller angle have been cloned. *LAZY1* (*LA1*), a novel grass-specific gene, is expressed during gravitropism sensitivity and plays negative role in polar auxin transport (PAT). Loss-of-function of *LA1* enhances PAT causing alteration of endogenous IAA distribution in shoots, leading to reduced gravitropism and a tiller-spreading phenotype of rice plants [12, 13]. *LPA1* (*Loose Plant Architecture 1*), identified on chromosome 5, encodes a plant-specific *INDETERMINATE DOMAIN* protein that regulates tiller angle by controlling the adaxial growth of tiller nodes [3]. *PIN-FORMED1* and *PIN-FORMED2* are auxin efflux transporters, and suppressing the expression of rice *PIN-FORMED1* or enhancing the expression of rice *PIN-FORMED2* can alter PAT and increase tiller angles [14, 15].

To further elucidate the genetic control of tiller angle, we used a RIL population that derived from a cross between a *japonica* and an *indica* rice cultivars to pinpoint two major QTLs for this trait. *qTAC8* and *qTAC9* were detected, and *qTAC8* was narrowed down to a 92 kb region where one candidate ORF was determined as the one encoding for *qTAC8*.

## Materials and methods

### Plant materials

A recombinant inbred line (RIL) population of 190 lines was generated from the cross between D50 and HB277 as described previously by Shao [16]. Briefly, one line (RIL-77) that carried the homozygous target segments RM339-RM210 on chromosome 8 and RM201-RM7306 on chromosome 9 from HB277 (S1A Fig) was chosen from the RILs to backcross with D50. The resultant BC_2_F_1_ was selfed to obtain a BC_2_F_2_ population containing 178 plants for genotyping and phenotyping, and a progeny (BC_2_F_2:3_) was used for phenotyping (S1B Fig). The RILs and BC_2_F_2_ population were grown at the experimental site of Fuyang district of Hangzhou in 2012, and the BC_2_F_2:3_ plants were grown at the experimental site of Lingshui (Hainan Province, China) and Fuyang district in 2013, respectively. Six plants per row were transplanted with a distance of 18 cm between plants within a row, and 18 cm between rows, and four rows were used to grow each line.

Two large segregating populations BC_2_F_4_ and BC_2_F_5_ carrying the heterozygous target segment RM339-RM210 on chromosome 8 and the D50 target segment RM201-RM7306 on chromosome 9, were picked for screening recombinant individuals in our target region. As a result, the homozygous recombinants were used for genotyping and phenotyping. One plant from BC_2_F_1_ carrying the heterozygous target segment RM339-RM210 and the HB277 target segment RM201-RM7306 was selected to consecutive backcross with D50 and produce BC_4_F_1_ and 120 BC_4_F_2_ individuals. Finally four NILs designated NIL-*qTAC8qtac9*, NIL-*qtac8qtac9*, NIL-*qTAC8qtac9* and NIL-*qTAC8qTAC9* were developed. This set of four NILs was planted in the experimental site of Lingshui in 2014 following a randomized block design with three repeats. Each line was grown in a six-row plot with 6 plants in each row and spacing of 18 x 18 cm.

### PCR and development of molecular markers

DNA was extracted following the protocols described by Murray and Rogers [17, 18]; Each 10 μL PCR reaction system contained 1 μL 10X PCR buffer, 1 μL dNTP (2 mM), 1μL of primer (1 mM), 0.1 μL Taq DNA polymerase (5 U/μL) and 1 μL template DNA. Polymerase chain reaction (PCR) comprised an initial denaturation step (95°C for 3 min), followed by 35 cycles of 95°C for 30 s, 55°C for 30 s and 72°C for 45 s, and ending with an extension step of 72°C for 10 min. PCR products were separated by electrophoresis and silver staining procedure. Simple sequence repeats (SSR) markers were selected covering the target region based on the published linkage map of rice (http://www.gramene.org). InDel (Insertion and Deletion) markers used for fine mapping of *qTAC8* were designed based on the reference Nipponbare and 93–11 genomic sequences.

### Sequencing and identification of candidate genes

The target gene in the candidate genomic region was predicted using the SIGnAL package (http://signal.salk.edu/). The full-length genomic DNA sequence of the candidate gene was determined by dividing it into several overlapping segments. Sequencing primers were designed according to the sequence of cv. Nipponbare in the target region. The PCR products were sequenced directly. Primer sequences used in this study are listed in S1 Table.

### RNA isolation and quantitative real-time PCR

Six plants of each line were pooled for RNA extraction. Total RNA samples from tiller base at four growth stages (seedling, tillering, heading and ripening stages) were extracted using RNAiso Plus (Takara), following the manufacturer’s instructions. The first cDNA strand was synthesized from 3 ug RNA using the First Strand cDNA synthesis Kit-Rever Tra Ace-α (ToYoBo). qRT-PCR analysis was performed on a Roche Light Cycler 480 device using various gene-specific primers. The rice *Ubi* gene (LOC_Os03g0234200) was chosen as reference gene. Reactions containing SYBR premix Ex TaqII (TaKaRa) were carried out in a final volume of 20 ul. The 2^-△△CT^ method [19, 20] was used to calculate relative levels of transcription. The PCR reaction implied an initial denaturation step (95°C for 4 min), followed by 50 cycles of 95°C for 15 s and 55°C for 30s. Three technical replicates were analyzed for each cDNA sample.

### Data analysis

Rice linkage maps were constructed using MAPMAKER/Exp Ver. 3.0, and genetic distances were converted into cM by using the Kosambi function. Composite interval mapping (CIM) analysis of QTL in the RILs and the BC_2_F_2_ population was performed with QTL cartographer Ver. 2.5 (statgen.ncsu.edu/qtlcart/WQTLCart.htm). QTLs were called where their LOD value exceeded 2.5. Mean phenotypic values were compared using the Student’s t test. Multiple comparison test and the correlation between genotypes and phenotypes were carried out by the SAS statistical software package.

## Results

### Primary mapping of tiller angle

**Fig 1:**
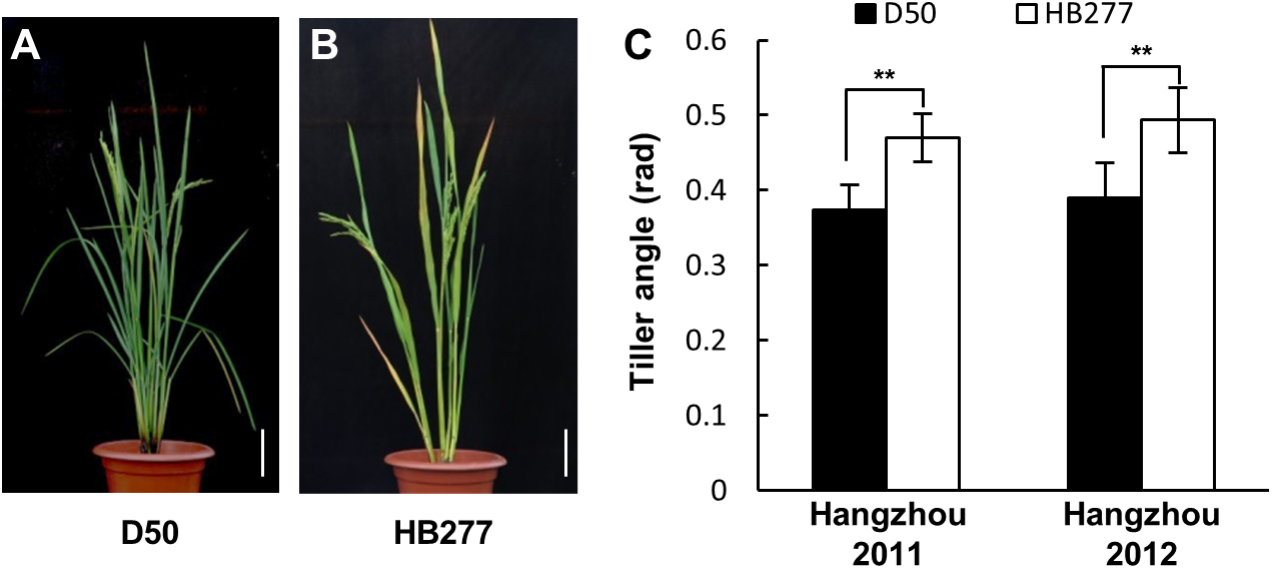
Variation in tiller angle between D50 and HB277. A and B, the phenotype of D50 and HB277 at heading stage. Scale bar 10 cm. C, mean tiller angle over two years. Error bars represent standard deviations (n = 6); ** indicates significant difference at P≤0.01.

Rice tiller angle serves as an important trait in rice. In this work, a glabrous *tropical japonica* rice D50 cultivar which exhibits relative compact plant type and an *indica* rice HB277 cultivar displaying a relative loose architecture were used in this study (Fig 1A and 1B). Then a RIL population was used to detect QTLs for this trait. Measurement of the tiller angle showed a continuous and normal distribution whose variation range was 0.267–1.010 rad (S2 Fig). QTL mapping strategy was conducted and two major QTLs for tiller angle named *qTAC8* on chromosome 8 and *qTAC9* on chromosome 9 were identified in this RIL population. *qTAC8* and *qTAC9* were mapped within the region of RM339-RM210 and RM201-RM7306, respectively. *qTAC8* could account for almost 33.4% of the variance, while *qTAC9* could explain around 17.4% of the variance in tiller angle (Table 1). The positive allele (increasing tiller angle) *qTAC9* was inherited from HB277. Unexpectedly, the positive allele *qTAC8* derived from D50 which displayed a relative compact plant architecture.

**Table 1:** Two major tiller angle QTLs detected by analysis of the RIL populations.

Year/Local	Locus	Chr	Interval	LOD value	Additive	R^2^ (%)
2011/Hangzhou	*qTAC8*	8	RM339-RM210	12.87	0.125	33.4
	*qTAC9*	9	RM201-RM7306	9.31	-0.086	17.4

### *qTAC9* is the same gene as *TAC1*

In accord to reports in the literature, we found that *qTAC9* was closely linked to *TAC1* flanked by the SSR loci RM201 and RM1026 [4], and a SNP in the 3’ splicing site of the fourth intron of *TAC1* was detected after sequencing the *TAC1* alleles in D50 and HB277 (S3A Fig). qRT-PCR analysis showed that the expression of *TAC1* in HB277 is significant higher than D50 (S3B Fig). These results coincided with the report that a mutation in the 3’ splicing site of the fourth intron from ‘AGGA’ to ‘GGGA’ can decrease the expression of *TAC1* and lead to a compact plant. Hence, we can anticipate that *qTAC9* and *TAC1* may correspond to the same gene.

### Characterization of *qTAC8*

**Fig 2:**
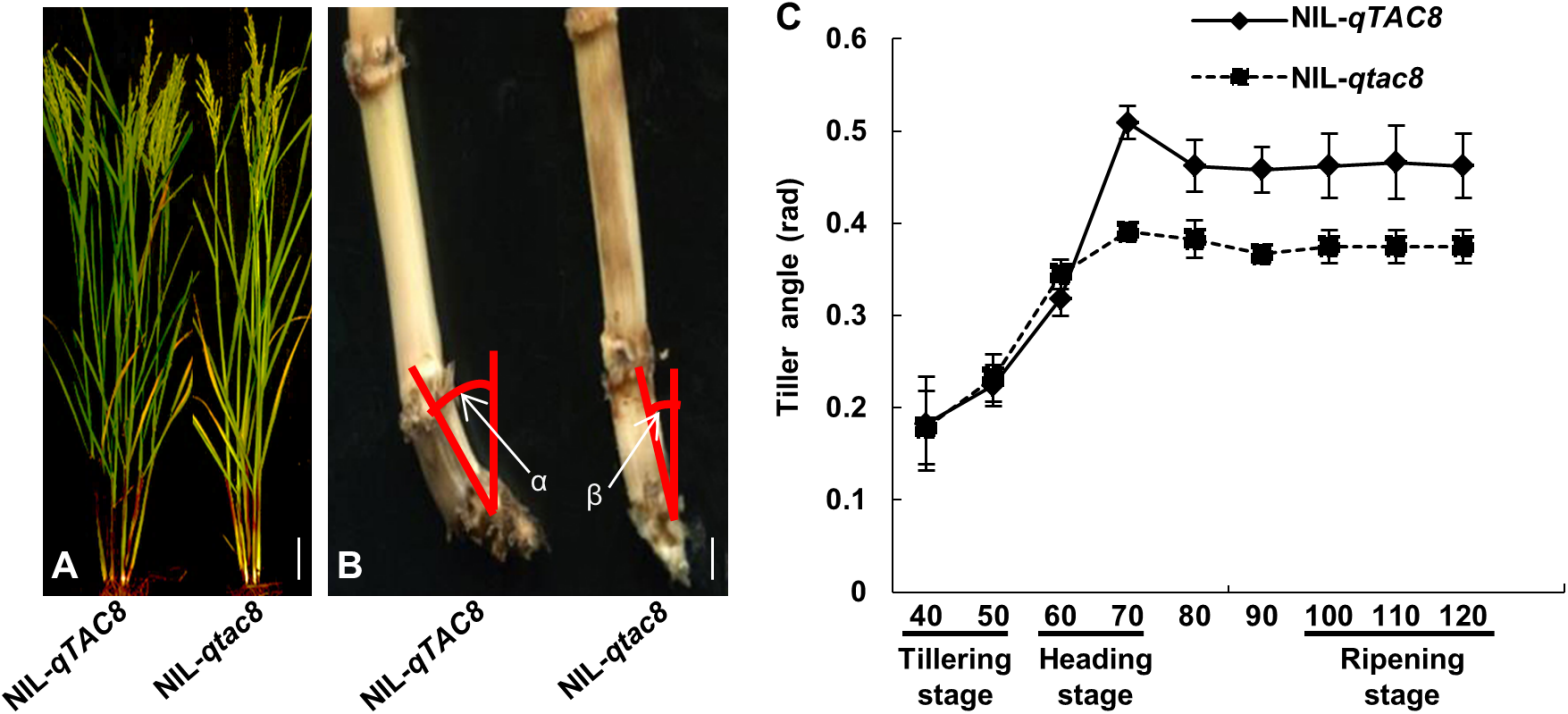
Characterization of NIL-*qTAC8* and NIL-*qtac8*. A, phenotypes of NIL-*qTAC8* and NIL-*qtac8* at ripening stage, respectively, Scale bar 10 cm; B, Comparison of the angle of the tiller base between NIL-*qTAC8* and NIL-*qtac8* at ripening stage, Scale bar 1 cm; C, tiller angle changing during development in NIL-*qTAC8* and NIL-*qtac8*. Error bars represent standard deviations (n = 6). The numbers of X-axis indicate the plant growing days after seed germination.

In order to investigate *qTAC8*, we measured the tiller angle of NIL-*qTAC8* and NIL-*qtac8*. The results showed that the tiller angle of NIL-*qTAC8* is significant larger than that in NIL-*qtac8* at the ripening stage, and the angle of tiller base in NIL-*qTAC8* is significant larger than that in NIL-*qtac8* (Fig 2A and 2B). We also found that the two NILs almost exhibited the same tiller angle at tillering stage, but NIL-*qTAC8* showed a greater tiller angle than that in NIL-*qtac8* since heading stage (Fig 2C). There were no significant differences in tiller numbers, plant height and spikelet fertility between NIL-*qTAC8* and NIL-*qtac8* (data not shown).

### Fine mapping of *qTAC8*

To further investigate the QTL *qTAC8*, a BC_2_F_2_ population containing 178 individuals was established (S1B Fig). The tiller angle of the BC_2_F_2_ population showed a normal distribution. According to the progeny test, the BC_2_F_2_ individuals could be classified into three subgroups of homozygotes for D50 (DD), for HB277 (HH) and heterozygotes at the targeted region. According to the tiller angle performance in the progeny test, paired t test was used to compare the difference between subgroups. Significant differences occurred between DD and the other two subgroups, HH and DH, suggesting that the DD genotype presented a larger tiller angle (0.462 rad) than HH (0.294 rad), while the tiller angle of the heterozygotes was an intermediate value (S4 Fig). These results showed that *qTAC8* is a QTL in D50 that presents a partial dominance and a positive additive effect.

**Fig 3:**
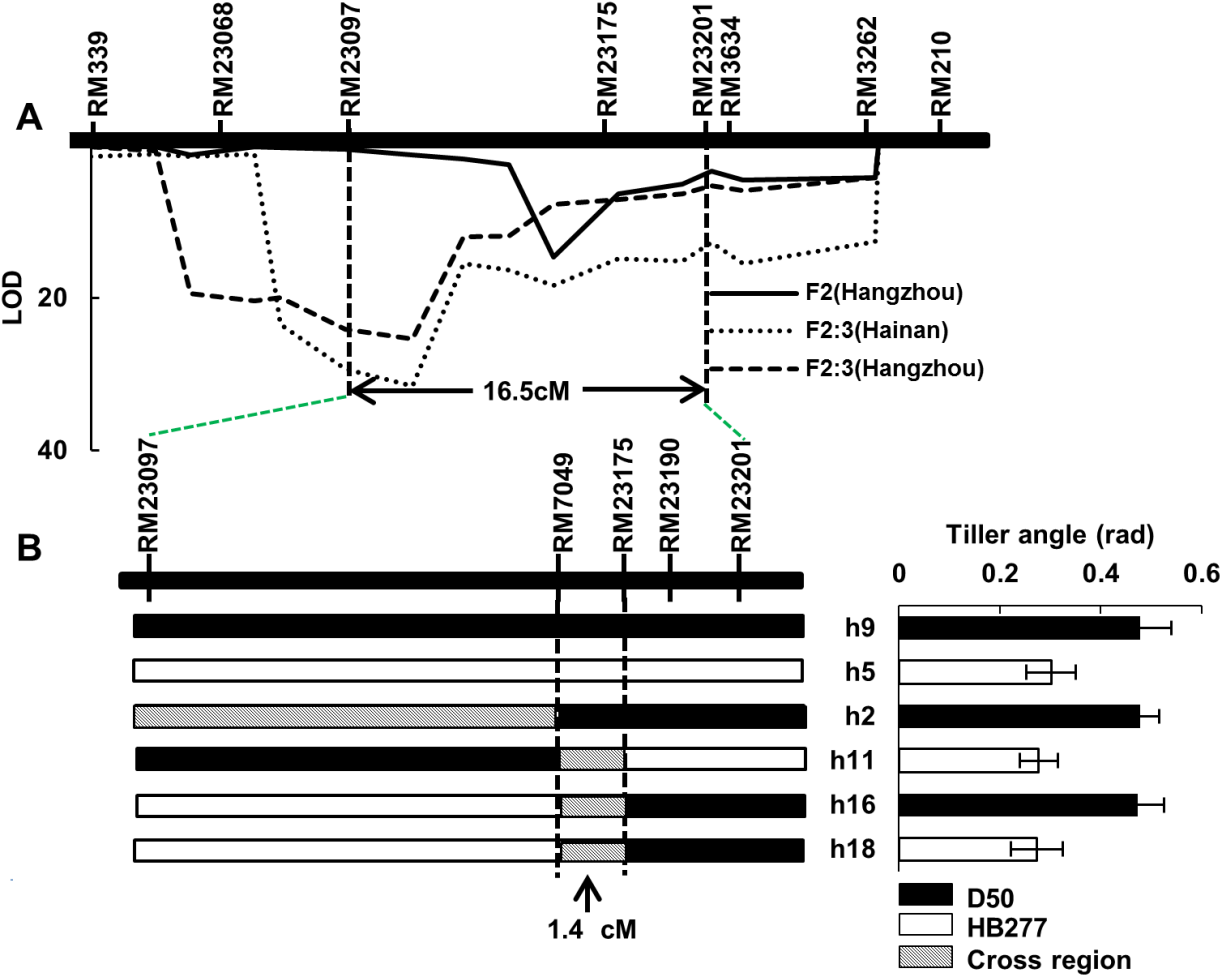
Preliminary mapping of *qTAC8*. A, *qTAC8* was mapped within the SSR loci RM23097 and RM23201 on chromosome 8 by using BC_2_F_2_ and BC_2_F_2:3_ populations. B, *qTAC8* was placed within a 1.4 cM region flanked by RM7049 and RM23175.

Seven more SSR markers were used to genotype the BC_2_F_2_ population and to construct a local linkage group covering 26.2 cM. The phenotype of two populations BC_2_F_2_ and BC_2_F_2:3_ were investigated in this study and *qTAC8* was mapped within a 16.5 cM interval flanked by RM23097-RM23201. *qTAC8* could account for 26.3% of the phenotype variance with additive effect of 0.07 rad in BC_2_F_2_ population, while in the BC_2_F_2:3_, the QTL could explain 47.1% and 51.8% of the variation in Hainan and Hangzhou, respectively (Table 2). In order to test and verify this, four informative homozygous recombinants were identified with four markers within RM23097-RM23201 and grouped into four genotypes according to the positions of recombinant breakpoints and allelic composition. Multiple comparisons between the tiller angle and recombinant individual genotypes, using the two non-recombinant lines as controls (h9 and h5), reflected that *qTAC8* was narrowed down to a 1.4 cM interval flanked by RM7049 and RM23175 (Fig 3A and 3B).

**Table 2:** *qTAC8* detected by analysis of the BC_2_F_2_ and BC_2_F_2:3_ populations.

Lines	Locus	Chr	Interval	LOD value	Additive	R^2^ (%)
BC_2_F_2_(Hangzhou)	*qTAC8*	8	RM23097-RM23201	14.58	0.070	26.3
BC_2_F_2:3_(Hainan)	*qTAC8*	8	RM23097-RM23201	31.50	0.076	47.1
BC_2_F_2:3_(Hangzhou)	*qTAC8*	8	RM23097-RM23201	25.50	0.062	51.8

**Fig 4:**
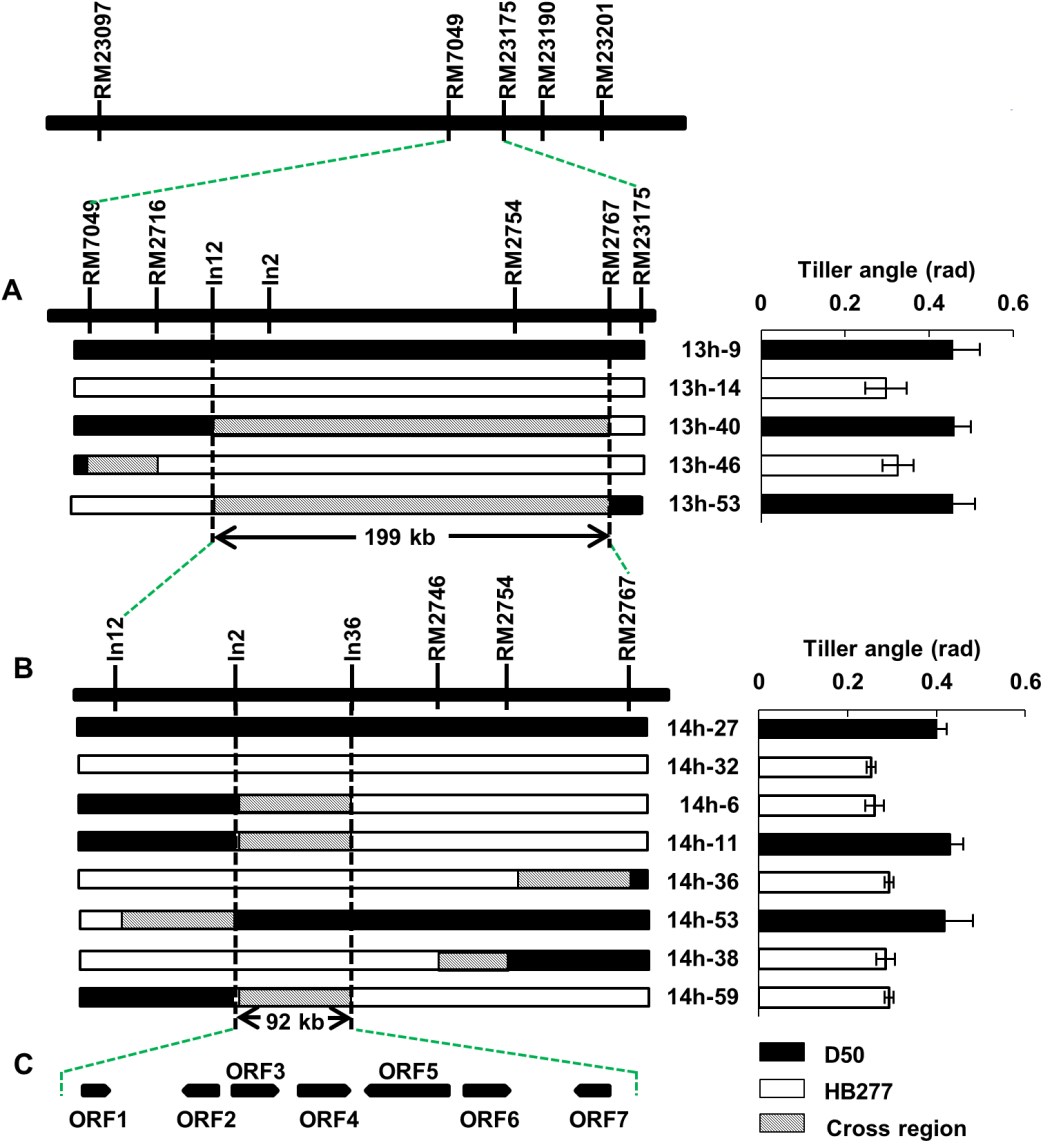
Fine mapping of *qTAC8*. A, Fine mapping of *qTAC8* within a 199 kb region flanked by In2 and RM2767 based on a separation population progenies. B, Fine mapping of *qTAC8* within a 92 kb region flanked by In2 and In36. C, Candidate genes for *qTAC8* within the region flanked by In12 and In36.

To further fine mapping *qTAC8*, a segregating population with 2,000 individuals derived from BC_2_F_4_ lines that carried a heterozygosis segments at the *qTAC8* region, were used to identify the recombinants between RM7049 and RM23175 (Fig 4A). Next, the identified recombinants were analyzed by seven more markers located between RM7049 and RM23175. Multiple comparisons were conducted and *qTAC8* was placed in a 199 kb region between In12 and RM2767. To position *qTAC8* more precisely, a large segregating population of 6,000 individuals was introduced from BC_2_F_5_, and a total of 40 recombinants were identified with the help of two markers In12 and RM2767, and four polymorphism markers within this region were developed to genotype these individuals. Multiple comparisons were also conducted between the genotypes of these homozygous recombinants and the phenotypes of their progeny. Finally, *qTAC8* was placed spanning on BAC P0431A03, in a 92 kb region flanked by In2 and In36 (Fig 4B**)**.

### Analysis of candidate genes for *qTAC8*

**Fig 5:**
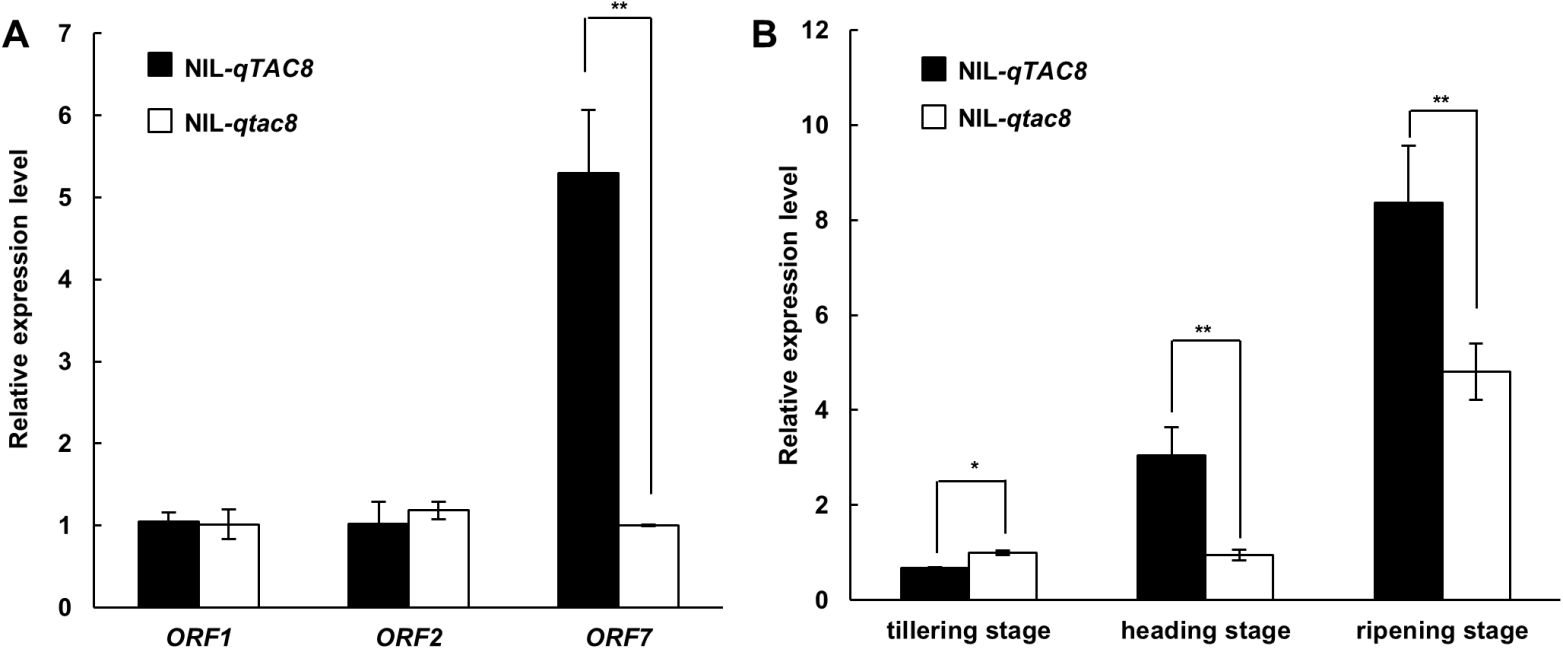
Relative expression analysis of *qTAC8* candidate genes. A, relative expression analysis of ORF1, ORF2 and ORF7. B, relative expression analysis of ORF7 at different developmental stages between the NIL pair. * indicate significant difference at level P≤0.05 (n = 3); ** indicate significant difference at level P≤0.01 (n = 3).

Based on the genome annotated database (http://signal.salk.edu/), the critical 92 kb region contains seven predicted ORFs. They encode a TCP family transcription factor, an ATP-dependent Clp protease adaptor protein, a transposon protein, two retrotransposon proteins, a zinc knuckle family protein and a basic helix-loop-helix protein (Fig 4C and Table 3). First, the transposon protein and retrotransposon proteins were excluded as candidates for *qTAC8*, leaving ORF1, ORF2, ORF6 and ORF7. Genomic sequencing of these four candidate genes in D50 and HB277 revealed that nucleotide diversity occurred except for ORF2. No products of ORF6 genomic DNA were identified in two parents (data not shown), which might be explained by a deletion of this gene during rice evolution, thus ORF6 was also excluded as candidate gene for *qTAC8* (data not shown). Quantitative real-time PCR was used to characterize the transcripts of the three candidate genes left in the tiller base at heading stage of NIL-*qTAC8* and NIL-*qtac8*. No significant difference in expression was observed for ORF1 and ORF2, while RNA expression level of ORF7 in NIL-*qTAC8* was significant higher than in NIL-*tac8* (Fig 5A). This result indicates that ORF7 is the possible candidate for *qTAC8*. Next we also found that the expression level of ORF7 was also increased at heading and ripening stages, but not at tillering stage, which coincides with the phenotypes of one NIL pair NIL-*qTAC8* and NIL-*qtac8* at three developing stages (Fig 2C and Fig 5B). All together, these data suggest that ORF7 is the candidate gene for *qTAC8*. Its cDNA stretched 1038 bp, comprises two exons, and encodes a 345 amino-acid protein containing a putative bHLH conserved domain. Although we found that seven single nucleotide polymorphisms (SNPs) occurred in ORF7 between the NIL pair, they didn’t cause any variation at the amino acid level. Further genomic sequencing results indicated that several nucleotides difference in the promoter and 3’ UTR region of ORF7 might be responsible for the alteration of gene expression (S5 Fig).

**Table 3:** The list of candidate genes between In2 and In36.

ORFs	Gene	Protein function
ORF1	Os08g33530	TCP family transcription factor, putative
ORF2	Os08g33540	ATP-dependent Clp protease adaptor protein ClpS containg protein, expressed
ORF3	Os08g33550	transposon protein, putative, Mutator sub-class
ORF4	Os08g33560	retrotransposon protein, putative, unclassified
ORF5	Os08g33570	retrotransposon protein, putative, unclassified
ORF6	Os08g33580	zinc knuckle family protein, putative
ORF7	Os08g33590	basic helix-loop-helix, putative, expressed

### Expression analysis other tiller angle-related genes

**Fig 6:**
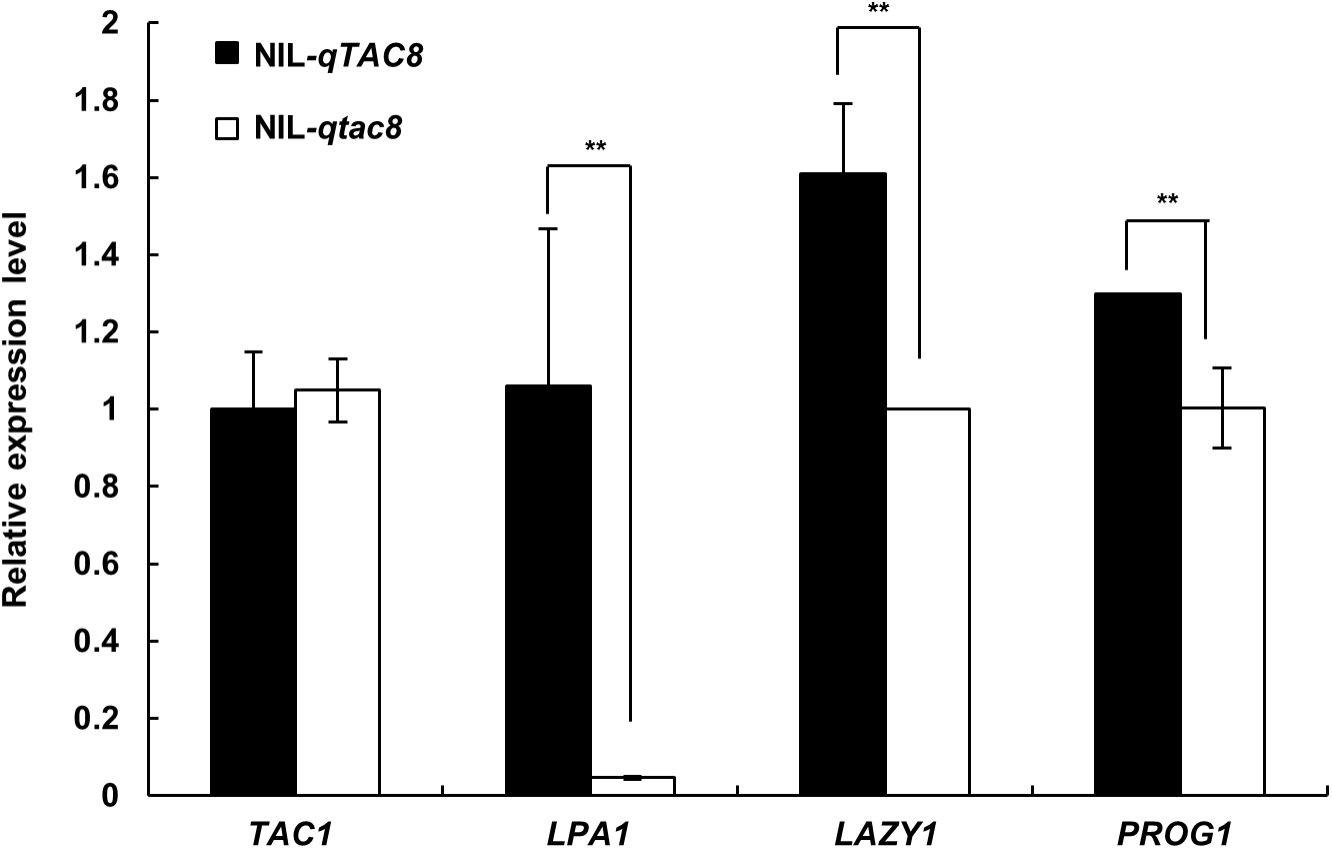
Expression analysis of tiller angle-related genes between NIL-*qTAC8* and NIL-*qtac8* at ripening stage. Values shown are mean ± SD (n = 3). ** indicate significant difference at level P≤0.01 (n = 3).

Tiller angle is known to be controlled by *TAC1*, *LPA1*, *LAZY1* and *PROG1*. To investigate the expression pattern of those genes in the NILs used in this study, qRT-PCR analysis was conducted. We found that *LPA1*, *LAZY1* and *PROG1* were all affected by the positive function of the *ORF7* allele, whereas there was no difference in expression of *TAC1* between the NIL pair (Fig 6).

### Genetic relationship between *qTAC8* and *qTAC9*

**Fig 7:**
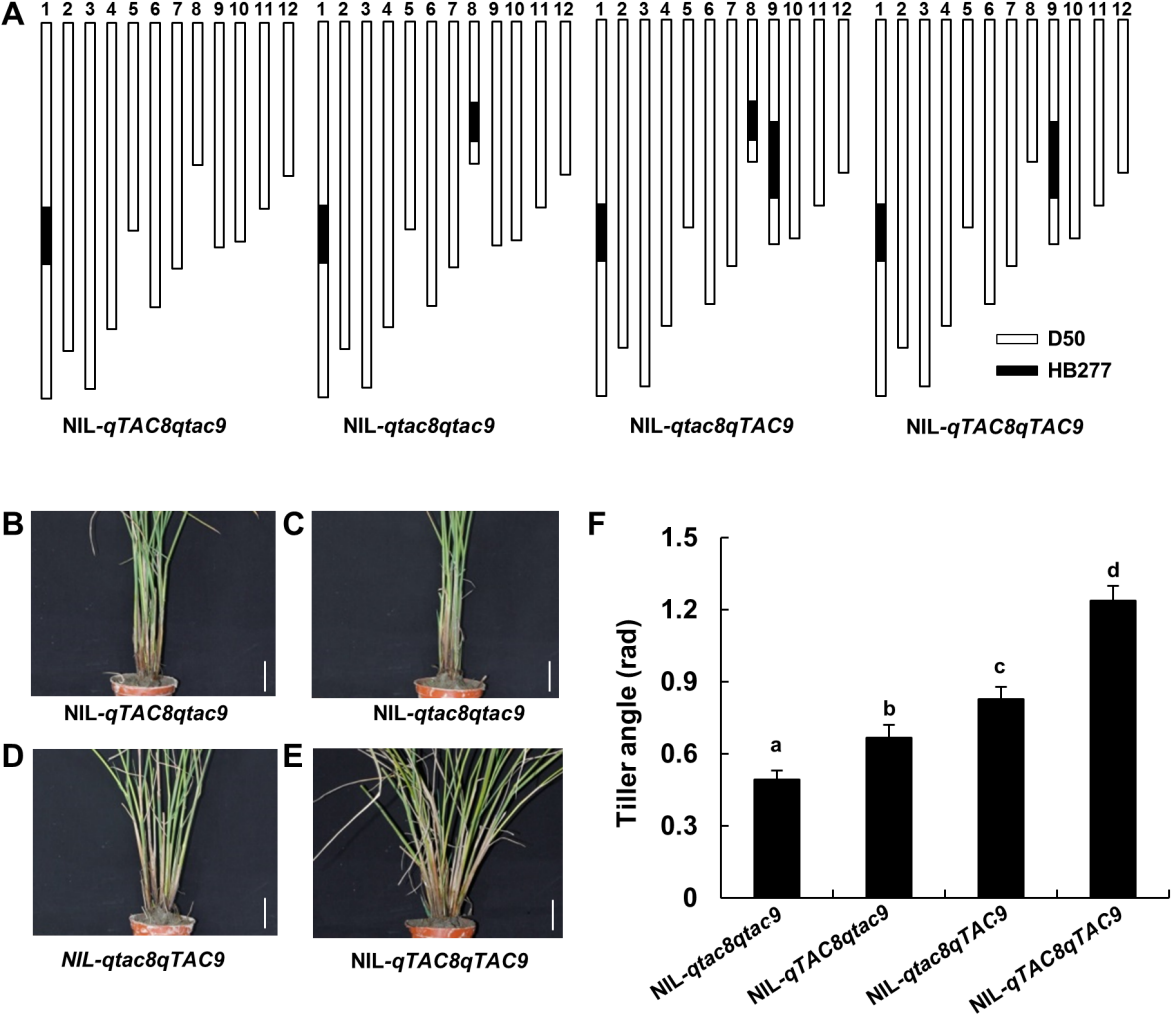
Genetic relationship between *qTAC8* and *qTAC9*. A, graphical genotypes of the four NIL pairs, including NIL-*qTAC8*qtac9, NIL-*qtac8qtac9*, NIL-*qtac8qTAC9* and NIL-*qTAC8qTAC9*. B-E, phenotypes of NIL-*qTAC8qtac9*, NIL-*qtac8qtac9*, NIL-*qtac8qTAC9* and NIL-*qTAC8qTAC9*, respectively. Scale bar 1 cm. F, Tiller angle of four NIL pairs. Error bars represent standard deviations (n = 6). Significant differences revealed by Tukey’s multiple comparison test are indicated by letters above bars (P < 0.05).

To further study the genetic relationship between *qTAC8* and *qTAC9*, a set of four NILs including NIL-*qTAC8qTAC9*, NIL-*qtac8qTAC9*, NIL-*qtac8qTAC9* and NIL-*qtac8qtac9* was produced to evaluate tiller angle (Fig 7A–7E). The results showed that the tiller angle of NIL-*qTAC8qTAC9* was significantly larger than in the other three NILs, with NIL-*qtac8qTAC9* and NIL-*qtac8*/*qtac9* presenting the smallest. The tiller angle of NIL-*qTAC8qTAC9* displayed an additive effect, indicating that *qTAC8* and *qTAC9* may be participating in different genetic pathways (Fig 7F).

## Discussion

Plant ideotype has been recognized as an advanced breeding concept and is regarded to be highly associated with high grain yield in rice breeding [21]. Rice traits for plant ideotype include plant height, stem strength, leaf morphology, panicle morphology, tiller angle among other critical traits. *Ideal Plant Architecture 1* (*IPA1*) was a major gene affecting rice productivity. Introduction of the *IPA1* semi-dominant gene into the *japonica* rice Xiushui 11 cultivar resulted in increased seed yield [22]. Tiller angle also plays a central role in rice production formation, and appropriate tiller angle is beneficial for improving rice production [3, 4]. Thus exploration of new genes controlling tiller angle would facilitate strategies to manipulate plant ideotype and increasing rice yield.

In this study, we identified two major QTLs controlling tiller angle on chromosomes 8 and 9 which were named *qTAC8* and *qTAC9*, respectively. Using an F_7_ RIL population derived from a cross between D50 and HB277 (Table 1), *qTAC9* was located between the SSR loci RM201 and RM7306 and the positive allele (increasing tiller angle) at *qTAC9* originated from HB277 with a loose plant architecture (Table 1). Previous studies revealed that the target region near *qTAC9* was a hot site controlling rice tiller angle on the long arm of chromosome 9 [1, 4, 6, 7], and one QTL named *TAC1* was isolated within this interval and cloned using a large F_2_ population derived from a cross between *indica* rice IR24 and an introgressiong line IL55 [4]. Sequencing analysis indicated that a SNP (‘AGGA’ to ‘GGGA’) occurred between D50 and HB277, and qRT-PCR analysis revealed that the RNA level of *TAC1* was significant reduced in D50 compared to HB277, suggesting that *qTAC9* was the same allele of *TAC1*, a gene that can form a plant with spread-out architecture and is wildly used within *indica* rice varieties (S3A and S3B Fig). A partially dominant gene *qTAC8* originated from D50 with a compact plant architecture and positive allele of the other QTL identified for tiller angle nearby. *qTA8-2* which controls rice tiller angle was firstly mapped between R1394 and RZ66 [1], which locates on the same region with *qTAC8*, suggesting that *qTA8-2* may be an allele of *qTAC8*. However primary QTL mapping, fine mapping, candidate gene prediction and qRT-PCR analysis indicated that ORF7, which encodes a basic helix-loop-helix protein, might be the gene underlying this QTL (Fig 4 and Table 3). Previous studies indicate that the semi-dominant gene *PROSTRATE GROWTH 1* which affects plant architecture, also encodes a basic helix-loop-helix transcriptional factor, suggesting that *qTAC8* might present a similar function as PROG1 [9, 10].

Analysis of genetic interactions among genes for tiller angle is required to better understand pathways controlling rice tiller angle formation. *qTAC8* (the positive allele from D50) and *qTAC9* (positive allele from HB277) are two semi-dominant genes for tiller angle, and a double mutant NIL-*qTAC8qTAC9* presented additive effect for that trait, suggesting that *qTAC8* and *qTAC9* are involved in different pathways. This was consistent with the similar expression of *TAC1* observed in NIL-*qTAC8* and NIL-*qtac8* (Fig 6 and Fig 7). Interesting, we found that *LAP1*, *LAZY1* and *PROG1* were all down-regulated in NIL-*qtac8* at ripening stage, implying that those genes might function in the same pathway with *qTAC8* (Fig 6). In addition, *qTAC8* encodes a predicted transcription factor, thus, whether *qTAC8* acts directly or indirectly on *LAP1*, *LAZY1* and *PROG1* remains to be investigated.

Gene expression in plants can be basically of two types, constitutive or with specific expression pattern. Although some genes can be expressed during the whole plant life, it may function during specific growth stages and contribute to a certain phenotype. The genetic control of tiller angle is very complex and phenotypes can change during different developmental stages. The *japonica* rice ZH11 is a typical rice cultivar which displays large tiller angle at the seedling and tillering stages but usually presents a more compact architecture after the heading stage. The rice mutants *lazy1* and *prog1* present a large tiller angle during all growing periods, while *lpa1* presents a large tiller angle at the seedling stage [3]. In this study, NIL-*qTAC8* and NIL-*qtac8* exhibit no differences in tiller angle at the tillering stage, but they show increasing *TAC8* expression and *TAC8* transcript levels are significant different during heading and ripening stages. It would be interesting to uncover the molecular mechanisms of how tiller angle is regulated during different rice development stages.

## Supporting information

S1 Fig Graphical genotypes of a RIL line and a BC_2_F_1_ line. A, a line derived from the RIL was used to backcross with D50 to obtain backcross populations. The circles indicate the region with QTLs for tiller angle; B, a BC_2_F_1_ line derived from RILs used for developing mapping population of *qTAC8*.
(TIF)

S2 Fig Tiller angle frequency distribution in the RIL population. (TIF)

S3 Fig Sequencing and relative expression analysis of *TAC1* allele in D50 and HB277. A, Sequencing analysis of *TAC1* allele; B, relative expression of the *TAC1* allele.
(TIF)

S4 Fig Tiller angle frequency distribution in BC_2_F_2_ population. The three genotypes of homozygous D50, HB277 and heterozygote at *qtac8* were identified by progeny test.
(TIF)

S5 Fig Sequencing of the *qTAC8* candidate genes *ORF7*. (TIF)

S1 Table Primer sequences used for this study. (DOCX)
