# Supplementary figures and images for: Fine mapping and candidate gene analysis of qTAC8, a major quantitative trait locus controlling tiller angle in rice (Oryza sativa L.)
Source: PLoS One. 2017 May 25;12(5):e0178177. doi: 10.1371/journal.pone.0178177 (PMC5444791; doi:10.1371/journal.pone.0178177)

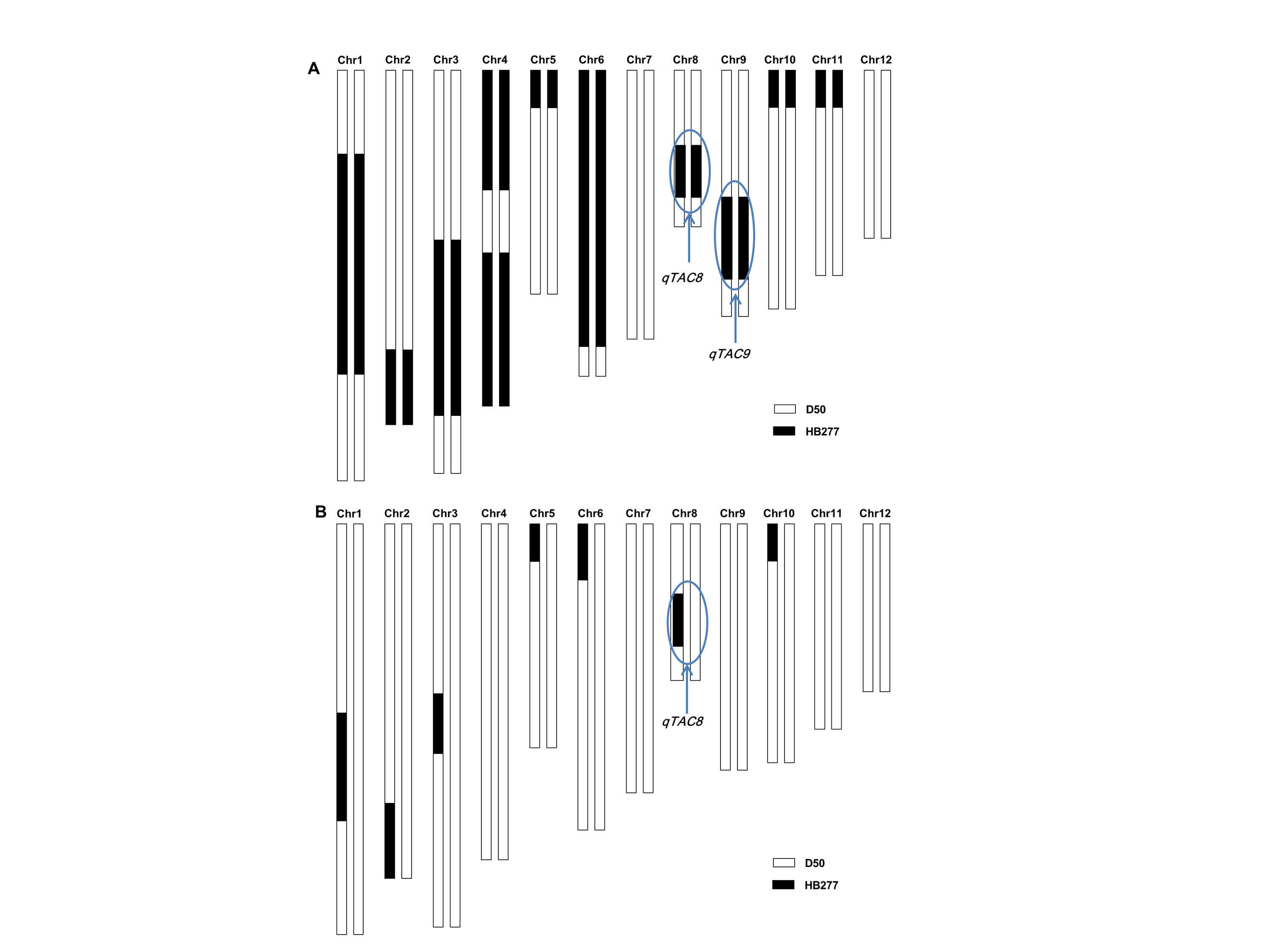

Supplement: S1 Fig — A, a line derived from the RIL was used to backcross with D50 to obtain backcross populations. The circles indicate the region with QTLs for tiller angle; B, a BC2F1 line derived from RILs used for developing mapping population of qTAC8. (TIF) [file pone.0178177.s001.tif]

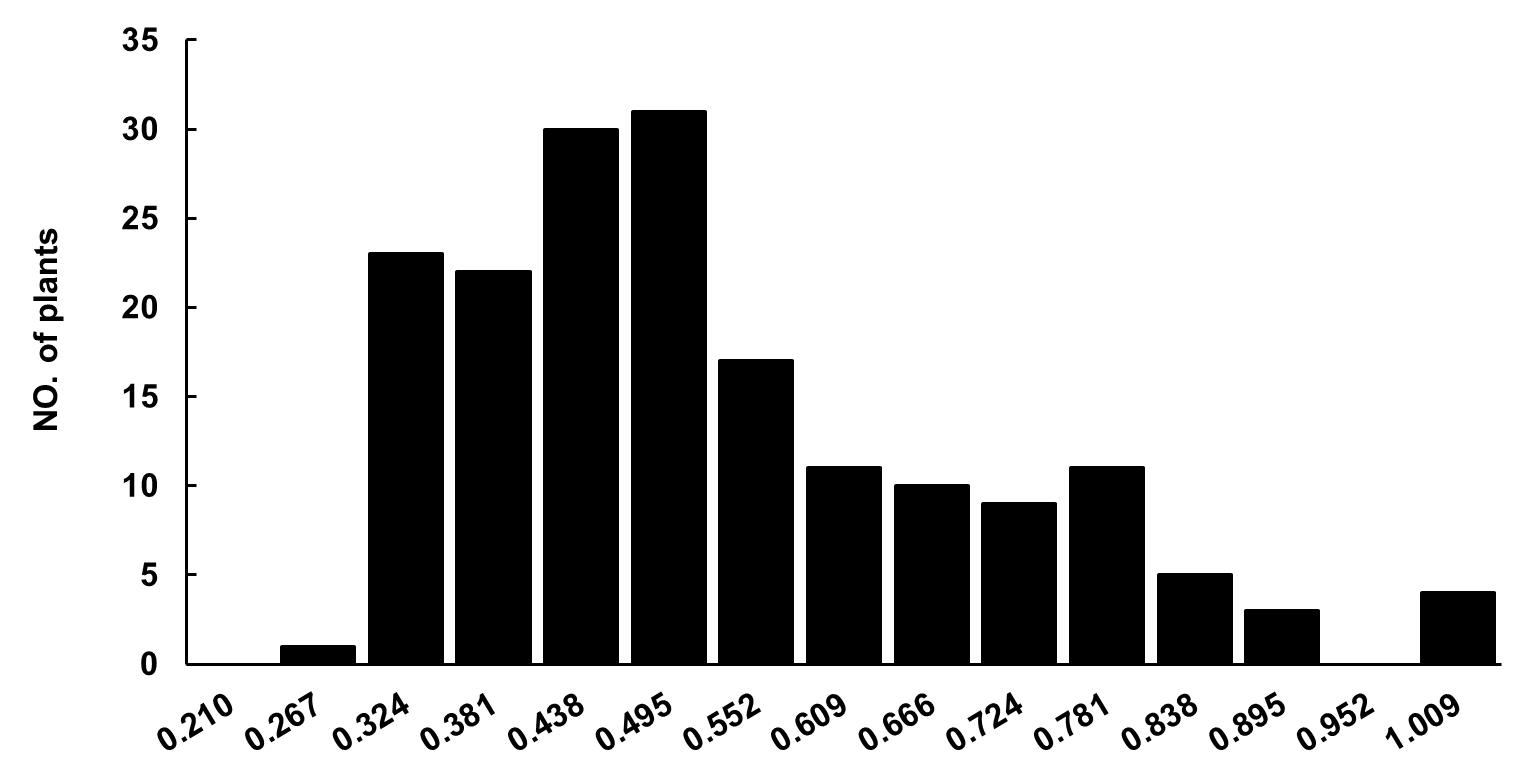

Supplement: S2 Fig — (TIF) [file pone.0178177.s002.TIF]

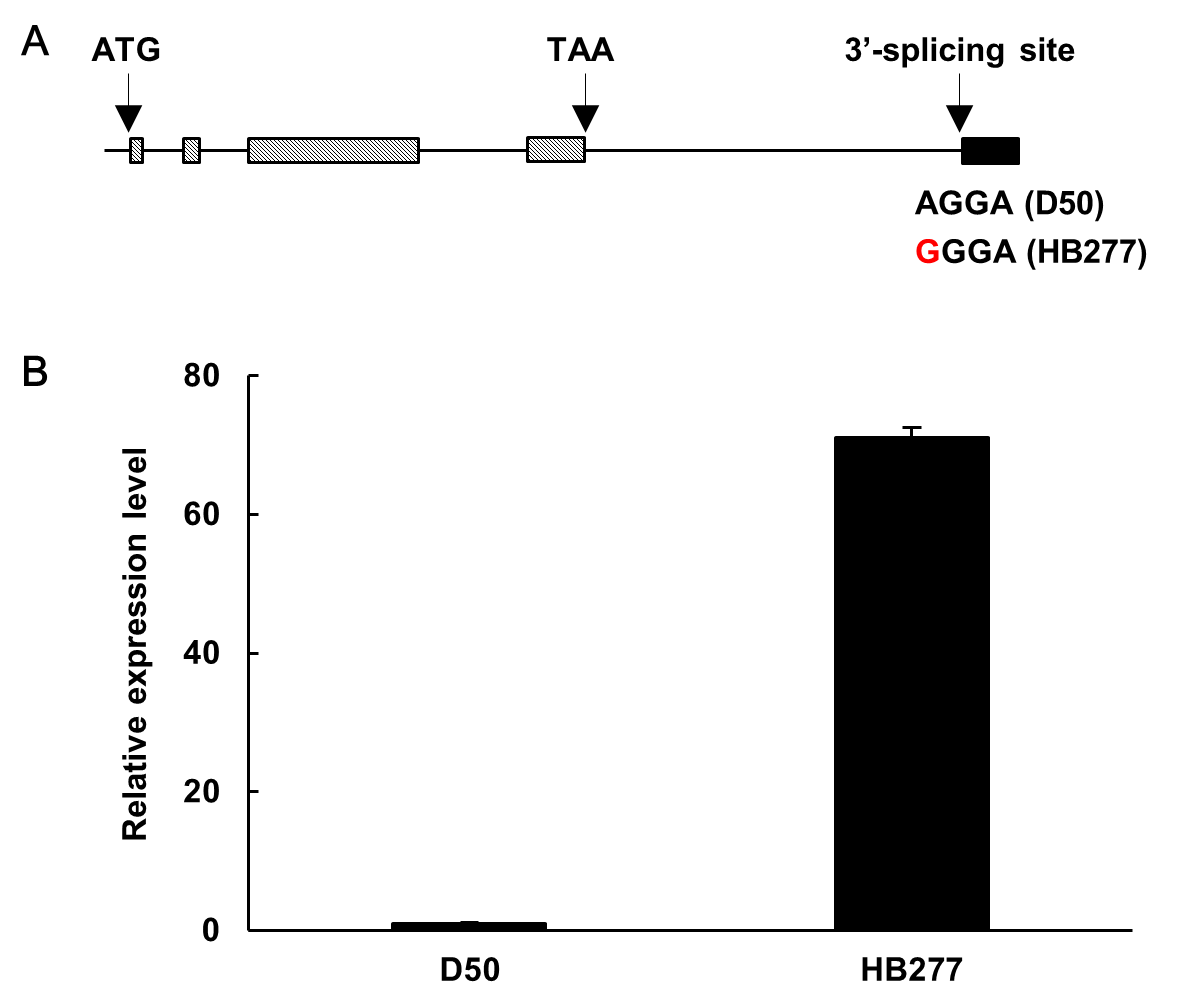

Supplement: S3 Fig — A, Sequencing analysis of TAC1 allele; B, relative expression of the TAC1 allele. (TIF) [file pone.0178177.s003.TIF]

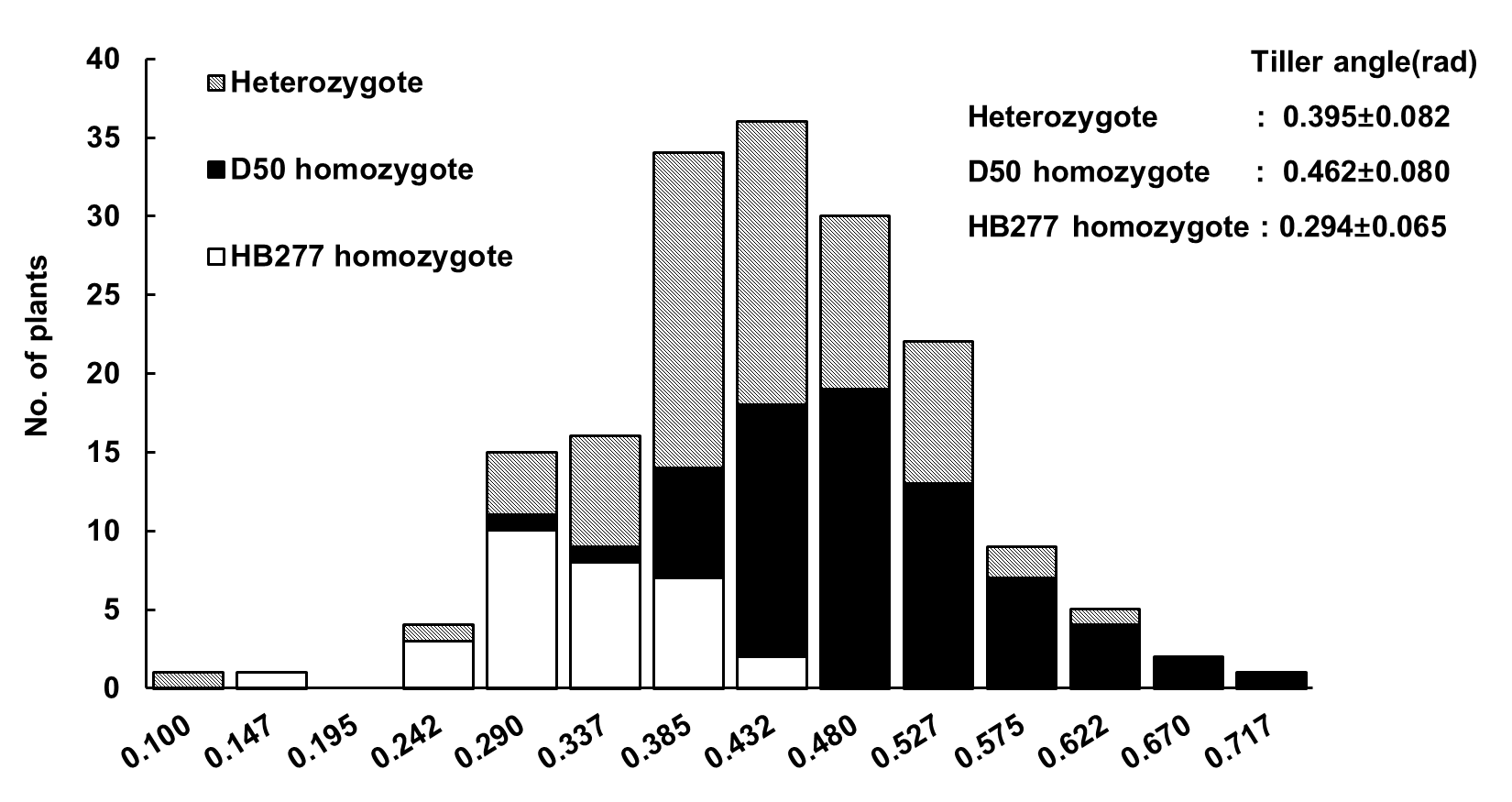

Supplement: S4 Fig — The three genotypes of homozygous D50, HB277 and heterozygote at qtac8 were identified by progeny test. (TIF) [file pone.0178177.s004.TIF]

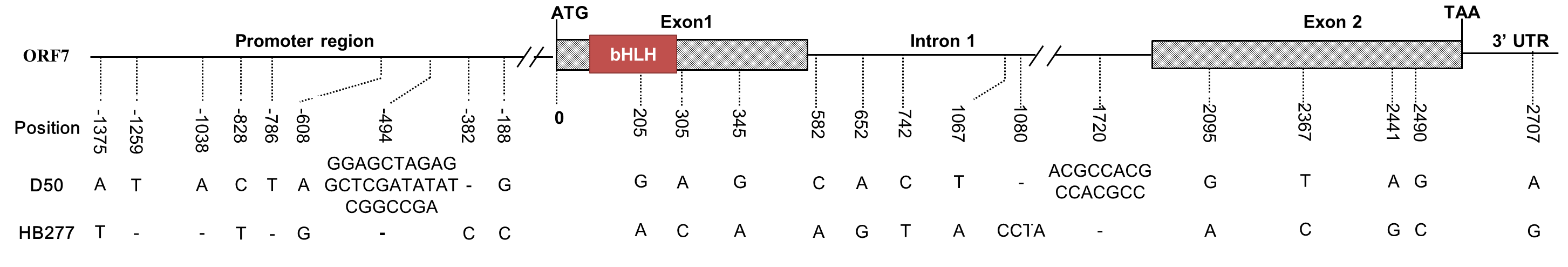

Supplement: S5 Fig — (TIF) [file pone.0178177.s005.TIF]
