# Supplementary material for: Fine mapping and candidate gene analysis of qTAC8, a major quantitative trait locus controlling tiller angle in rice (Oryza sativa L.)
Source: PLoS One. 2017 May 25;12(5):e0178177. doi: 10.1371/journal.pone.0178177 (PMC5444791; doi:10.1371/journal.pone.0178177)
Supplement: S1 Table — (DOCX) [file pone.0178177.s006.docx]

**S1 Table** Primer sequences used for this study

| Primer name | Forward (5'-3') | Reverse (5'-3') |
| --- | --- | --- |
| For mapping | | |
| Indel12 | CAAACGAACTAAGGGTGCG | GGAATACGATGGGGCTTTC |
| Indel2 | CGGTAATAAGATAAGATCC | GTGTCCCATTATAGAGTTG |
| Indel36 | TAGGGCTCCTCAACTCA | CAAGCGTCATTATACTATCTG |
| RM2746 | CGAACCTAAAGGTTGGCATT | ATGCTTGGGACGGTGATAAA |
| RM2754 | AGAAGAAGTAGCACTCGCCG | TCCCATGTATGCCCTAGCTC |
| RM2767 | TCCTTGTGAAAGAGGAGAGAGC | AGATGAGGCCGGAAGTGTC |
| For sequencing | | |
| S*ORF1* | AGTGACGGCAATGGTAG | CCTTTACTGGCTTTACTCAT |
| S*ORF2* | TCCATTATTAGTCCATCG | AATCTTGAAATCCGTTTG |
| S*ORF7-1* | CGACCACCACTACCACT | TTTGCTCGTACCACTTCT |
| S*ORF7-2* | CCCTAACCAAACTGGGATG | GCGAGGATGGAGGAAAA |
| S*ORF7-3* | TACCAAAGCCAAAAGC | TCAAAGCACCCAACAC |
| For real-time RT-PCR | | |
| *Ubiquitin* | GCTCCGTGGCGGTATCAT | CGGCAGTTGACAGCCCTAG |
| *ORF1* | CGCCATCAACCGCCTCA | GCCTCCCCTTTCTCCTTCG |
| *ORF2* | ATCGAGTCCTCCTCCACAA | CCACTGACAGCCCGTTT |
| *ORF7* | ACGACGGCGAACACGGCTAC | TGCCTCTTGATGCTCCCACC |
| *TAC1* | GAGATGGCTCTAAAGGTGTTC | CGTGCCAATTGCAAGTATACC |
| *LAZY1* | GCAACGCCGAGATGAACG | ATAATTCCAGCACCAAGTAGTCG |
| *LPA1* | GCGTATGTATGTAAAGCAAG | GAAACGACCTACGAAACTAC |
| *PROG1* | ATGCCCATCGCCTCCCAT | GCCGAGCTCGAGGACAAG |
